# Application of Biochar-Immobilized *Bacillus megaterium* for Enhancing Phosphorus Uptake and Growth in Rice

**DOI:** 10.3390/plants14020214

**Published:** 2025-01-14

**Authors:** Keru Yu, Zhenyu Wang, Wenyan Yang, Shuai Li, Dongtao Wu, Hongtao Zheng, Zhengqian Ye, Shaona Yang, Dan Liu

**Affiliations:** 1 State Key Laboratory of Subtropical Silviculture, Zhejiang A&F University, Hangzhou 311300, Chinam17866926886@163.com (Z.W.); ls1851325778@163.com (S.L.); m18376727861@163.com (H.Z.); yezhq@zafu.edu.cn (Z.Y.); 2 School of Environmental and Resources, Zhejiang A&F University, Hangzhou 311300, China; 3 Key Laboratory of Soil Contamination Bioremediation of Zhejiang Province, Zhejiang A&F University, Hangzhou 311300, China; 4 College of Tea Science and Tea Culture, Zhejiang A&F University, Hangzhou 311300, China; wyyang1808@163.com; 5 Soil Fertilizer and Plant Protection and Energy Sources Station of Lishui City, Hangzhou 323000, China; wudongtao1@163.com; 6 Zhoushan Agricultural Technology Extension Center, Zhoushan 316021, China

**Keywords:** rice husk biochar, phosphate solubilizing bacteria, phosphorus gene community, microbial catabolic activity, phosphorus content

## Abstract

Phosphorus (P) is an essential nutrient for rice growth, and the presence of phosphate-solubilizing bacteria (PSB) is an effective means to increase soil P content. However, the direct application of PSB may have minimal significance due to their low survival in soil. Biochar serves as a carrier that enhances microbial survival, and its porous structure and surface characteristics ensure the adsorption of *Bacillus megaterium*. Inoculating rice husk biochar-immobilized with *Bacillus megaterium* (BMB) resulted in dissolved inorganic and organic P levels of 39.55 and 31.97 mL L^−1^, respectively. Subsequently, rice pot experiments were conducted to investigate the response of soil microbial P mobilization and P uptake in rice to fertilizer inputs. The organic fertilizer (OF) combined with BMB treatment (MOF) showed the highest soil available phosphorus (AP) at 38 days, with a value of 7.83 mg kg^−1^, as well as increased the *pqqC* abundance while decreasing the abundance of *phoD* bacterial communities compared with the control. Furthermore, the bioavailable P reservoir (H_2_O–Pi and NaHCO_3_–Pi) in soil was greatly increased through the fertilizer input and microbial turnover, with the highest H_2_O–Pi (3.66 mg kg^−1^) in OF treatment and the highest NaHCO_3_–Pi (52.65 mg kg^−1^) in MOF treatment. Additionally, carbon utilization analysis was applied using the commercial Biolog system, revealing that the MOF treatment significantly increased the utilization of carbohydrates, polymers, and amino acid carbon sources. Moreover, compared to the control, MOF treatment significantly increased the shoot (0.469%) and root P (0.516%) content while promoting root development and thereby supporting rice growth. Our study demonstrates that the MOF treatment displayed higher P levels in both soil and rice plants, providing a theoretical basis for further understanding the role of biochar-based bacterial agents in rice P management.

## 1. Introduction

Rice is a major food source, particularly in Asia [1], with adequate phosphorus (P) being essential for its root development and maturation [2]. However, soil pH, mineral composition and other physiochemical factors can limit phosphorous availability [3]. As a result, rice cultivation practices often rely on the application of large amounts of phosphorus fertilizer to ensure that it does not become a limiting factor in rice production. However, PO_4_^3−^ is easily fixed by soil particles, making it difficult for crops to absorb and utilize, leading to low phosphorus utilization efficiency [4]. Furthermore, long-term application of phosphorus fertilizer not only results in the wastage of this non-renewable mineral resource [5] but also in soil phosphorus pollution, soil degradation, compaction and eutrophication of water bodies [6]. Therefore, it is crucial to activate immobilized phosphorus in paddy soil to enhance the efficiency of rice phosphorus utilization.

Phosphate-solubilizing bacteria (PSB) are widely used in agriculture as a biofertilizer to improve phosphorus absorption and crop yields [7]. These bacteria have the ability to secrete organic acids, which can chelate Ca, Fe, and Al, leading to the release of phosphorus [2]. Specifically, PSB expresses pyrroloquinoline quinone (PQQ), a key enzyme in gluconic acid synthesis that promotes the release of inorganic phosphorus (Pi) [8]. The *pqqC* gene is commonly used as a bioindicator for the presence of Pi-solubilizing microorganisms and plays an important role in this process [9]. Moreover, microorganisms are essential in facilitating the accumulation of phosphorus in plants by mineralizing organic phosphorus (Po) via enzyme action [10]. Detection of *phoD* gene encoding alkaline phosphatase is also used to assess changes in bacterial communities driving Po mineralization [8,11]. Inoculating PSB is considered to be an effective way to improve soil available phosphorus (AP) content and achieves high phosphorus nutrient efficiency. However, practical application is hindered by the low survival rate of these bacteria, limiting the effectiveness of PSB’s benefits [12]. Therefore, finding a highly stable and robust carrier material could enhance the overall availability of phosphorus nutrients.

Biochar, a carbon-rich material produced by the pyrolysis of biomass under anaerobic conditions [13], is applied as a soil amendment to enhance phosphorus cycling in soil–plant systems [14]. It is widely recognized as an excellent carrier material due to its unique physical and chemical characteristics that provides a protective habitat for inoculated microbial communities and have shown positive effects on increasing microbial abundance in soils [15,16]. Previous studies have demonstrated that the porous structure of biochar enhances PSB adsorption capacity [17]. Furthermore, biochar contains a large amount of nutrients such as organic carbon, phosphorus and nitrogen, along with numerous high-energy adsorption sites that provides substrates and reaction sites for bacteria immobilization [18,19].

Based on our previous soil incubation experiment, we found that the combination of 1% (*w*/*w*) biochar-immobilized *Bacillus megaterium* (BMB) and 0.1% OF resulted in higher soil AP compared to the combination of 0.5% BMB and 0.1% OF. In this study, rice plants were cultivated in the presence of either 1% Rice husk biochar (RHB) or BMB and 0.1% OF, which were applied to the soil in order to investigate: (1) responses of different phosphorus fractions to different fertilization regimes (2) responses of bacterial communities harboring *pqqC* and *phoD*, as well as their associated carbon metabolism (3) relationships among soil properties, phosphorus fractions, *pqqC*- and *phoD*-harboring bacterial communities, and metabolic activity of carbon substrate and (4) factors influencing phosphorus uptake in rice. We hypothesized that different fertilization regimes would alter soil phosphorus fractions and accelerate its conversion via the abundance of the inoculated microorganisms. An increase in AP content and uptake results in increased rice growth. The findings of this study could contribute to a better understanding of the mechanisms involved in soil P cycling, as well as provide a theoretical basis for improving the utilization rate of P in paddy soil and promoting rice growth.

## 2. Results

### 2.1. Characterization Analyses of RHB and BMB

SEM images revealed that the RHB surfaces were predominantly microporous, with *Bacillus megaterium* appearing as an elliptical rod-like structure distributed on the biochar surface. Interestingly, no obvious surface structure alterations were observed on the biochar due to its association with bacteria (Figure S1a,c). EDS images indicated that RHB primarily consisted of biomass material composed of C and O, along with associated N, K, Ca, S and P elements. Moreover, loading of the bacteria (BMB) resulted in decreases of each element within the biochar material (Figure S1b,d).

Our RHB and BMB samples were analyzed using FTIR, indicating a high degree of similarity in terms of functional groups (Figure S2). Both exhibited characteristic peaks at 3423 and 3425 cm^−1^ (–OH stretching vibrations) and 2815 and 2814 cm^−1^ (asymmetric stretching vibrations of –CH_2_ in fatty acids), respectively. The presence of peaks at 1593 cm^−1^ indicated the existence of Carboxyl (C=O) groups, while multiple absorption peaks at 680–1450 cm^−1^ were also observed (Figure S2). These data suggested that bacterial inoculation did not alter the chemical composition or structure of the biochar.

### 2.2. Changes in BMB Phosphorus Solubilizing Capacity

We then investigated whether BMB samples exhibited significant ability to mobilize phosphorus (Figure S3). In the inorganic phosphorus medium, the AP content increased rapidly during the first 1–2 d after inoculation and reached its peak on day 3 (29.55 mg L^−1^), remaining relatively stable at later stages of culture. Additionally, BMB demonstrated considerable solubilizing capacity for organic phosphorus. The AP content rose rapidly from days 1 to 3, reaching its maximum on day 4 (31.97 mg L^−1^), and then stabilized.

### 2.3. Changes in Soil Properties

We initially investigated changes in soil properties over time (Table S2). The data indicated that within the first 24 h (day 0), the soil pH, AP and TP increased in all treatments compared to the control. The results showed that the experimental treatment had a significant effect on soil pH value and phosphorus content in the short term, which may be caused by additives. At day 21, the MOF combination treatment led to the greatest increase in soil pH (6.63) and AP content (14.46 mg kg^−1^) and was significantly higher than the control. Additionally, soil acid phosphatase activity in the OF treatment significantly increased compared to other treatments (*p* < 0.05). At day 38, all treatments enhanced soil pH, AP content and acid phosphatase activity compared to the control. Notably, the MOF treatment exhibited significant increases in pH (6.19) and acid phosphatase activity (0.282 mg g^−1^·24 h) (*p* < 0.05).

### 2.4. Changes in Soil Phosphorus Fractionation

**Figure 1:**
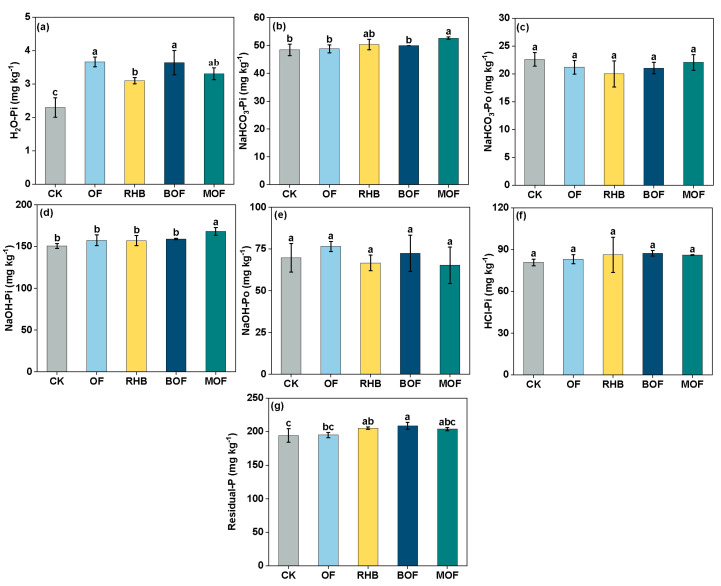
Content of different phosphorus fractions in soil on day 38. (**a**) H_2_O–P (**b**) NaHCO_3_–Pi (**c**) NaHCO_3_–Po (**d**) NaOH–Pi (**e**) NaOH–Po (**f**) HCl–P (**g**) Residual–P. Error bars represent the standard deviation of the mean (n = 3). Different letters within a column indicate significant differences at *p* < 0.05. CK, control without fertilizer; OF; 0.1% organic fertilizer; RHB, 1% rice husk biochar; BOF, 1% rice husk biochar and 0.1% organic fertilizer. MOF, 1% rice husk biochar-immobilized Bacillus megaterium (BMB) and 0.1% organic fertilizer.

The presence of phosphorus in the soil after 38 d indicated that all treatments significantly increased levels of H_2_O–P (*p* < 0.05) compared to the control, with increases of 35.03–59.60% (Figure 1a). The MOF treatment showed significantly higher levels of NaHCO_3_–Pi (52.65 mg kg^−1^) and NaOH–Pi (168.15 mg kg^−1^) compared to the control (*p* < 0.05), with increases of 8.71% and 11.61%, respectively (Figure 1b,d). There was no significant difference in NaHCO_3_–Po, NaOH–Po and HCl–Pi content among the different treatments (*p* > 0.05) (Figure 1c,e,f).

### 2.5. Response of the Soil pqqC- and phoD-Harboring Bacterial Communities

The abundance and diversity of *phoD*- and *pqqC*-harboring communities were influenced by the different fertilization regimes (Figure S4). Compared to the control, the Shannon diversity index of the *pqqC* community was significantly changed in the OF treatment, which increased by 22.38% (Figure S4a). Additionally, the Chao1 abundance index of the *pqqC* community was significantly affected in both the BOF and MOF treatments, increasing by 55.69% and 50.25%, respectively (*p* < 0.05) (Figure S4b). Compared with the control, all treatments decreased the Shannon diversity and Chao1 abundance indices of *phoD* communities, with reductions ranging from 3.38–12.29% and 18.98–36.14%, respectively, while BOF treatment decreased significantly (Figure S4a,b).

**Figure 2:**
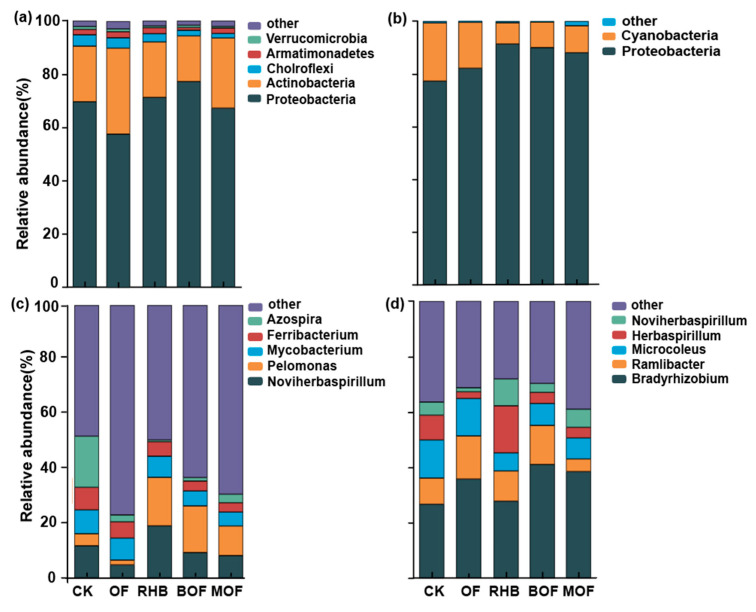
Relative abundance at the phylum and genus levels in (**a**,**c**) *pqqC* and (**b**,**d**) *phoD* community composition for different treatments. Abundance > 0.5% for phyla and > 1.0% for genus, respectively. CK, control without fertilizer; OF; 0.1% organic fertilizer; RHB, 1% rice husk biochar; BOF, 1% rice husk biochar and 0.1% organic fertilizer. MOF, 1% rice husk BMB and 0.1% organic fertilizer.

Under all fertilization treatments, BOF and RHB treatments showed the maximum relative abundance of the dominant phylum *Proteobacteria* in the bacterial community carrying *phoD* and *pqqC* genes, which was 77.20% and 91.37%, respectively. (Figure 2a,b). In addition, *Actinobacteria* accounted for a considerable proportion of the *pqqC* gene-carrying bacterial communities. Especially under the treatment OF, the relative abundance of *Actinobacteria* reached the highest value, 32.31% (Figure 2a). The predominant genera across all samples were *Noviherbaspirillum* (4.75–11.75%), *Pelomonas* (1.73–17.15%) and *Mycobacterium* (5.12–12.08%) for the *pqqC* community (Figure 2c). RHB showed the highest abundance of *Noviherbaspirillum* and *Pelomonas*, while the MOF treatment showed the lowest abundance of *Mycobacterium* (Figure 2c). Moreover, *Bradyrhizobium* (26.73–41.40%), *Ramlibacter* (4.53–15.58%), and *Microcoleus* (6.52–13.76%) predominated in the *phoD* communities. The BOF treatment exhibited the highest abundance of *Bradyrhizobium* (41.40%), whereas the OF treatment possessed the highest abundance of *Ramlibacter* (15.58%) compared to other treatments (Figure 2d).

### 2.6. Response of the Soil Microbial Activity

**Figure 3:**
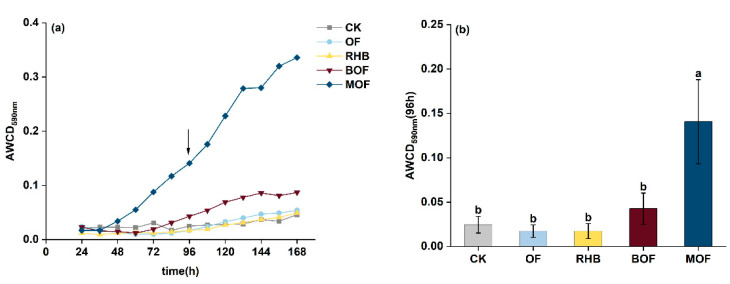
Dynamics of carbon source utilization in EcoPlates. (**a**) Average well color development (AWCD) index of the soil samples and (**b**) Total AWCD after 96 h. Error bars represent the standard deviation of the mean (n = 3). Different letters within a column indicate significant differences at *p* < 0.05. The black arrow indicates the sampling time (96 h) chosen for further analysis. CK, control without fertilizer; OF; 0.1% organic fertilizer; RHB, 1% rice husk biochar; BOF, 1% rice husk biochar and 0.1% organic fertilizer. MOF, 1% rice husk BMB and 0.1% organic fertilizer.

The effects of different treatments on soil microbial activity were assessed based on the level of color development (AWCD) in the Biolog plates. Changes in AWCD over the 168 h incubation period revealed distinct differences between MOF and the other treatments, with these variances becoming more pronounced over time (Figure 3a). Following a 96 h incubation, MOF treatment showed a significant increase in total AWCD for all 31 carbon substrates (*p* < 0.05), with a 4.70-fold rise in total AWCD when compared with the control (Figure 3b). Specifically, the MOF treatment samples significantly increased the utilization of carbohydrates, polymers, and amino acids, while BOF and MOF treatments showed significant increases in the utilization of carboxylic acids. And there were no significant differences in the utilization of Phenols or Amines (*p* > 0.05) (Figure S5).

**Figure 4:**
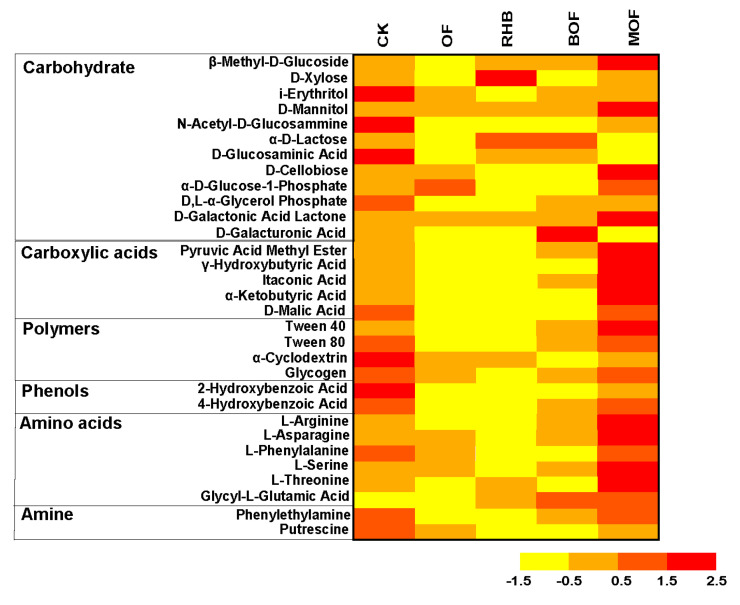
Biolog EcoPlate substrate utilization (Abs 590 nm) after 96 h of incubation. CK, control without fertilizer. OF, 0.1% organic fertilizer. RHB, 1% rice husk biochar. BOF, 1% rice husk biochar and 0.1% organic fertilizer. MOF, 1% rice husk BMB and 0.1% organic fertilizer.

The utilization of the 31 carbon substrates after 96 h incubation differed among our experimental treatments, with the biggest differences observed between the MOF and control (Figure 4). The utilization of carboxylic acids and amino acids in MOF treatment was much higher than that in other treatments. In addition, the utilization efficiency of carbohydrates (β-methyl-D-glucoside, D-mannitol, D-cellulobiose and D-galactolactone) also reached the highest in MOF treatment. Moreover, i-erythritol, N-acetyl-D-glucosammine, α-cycogen, and 2-hydroxybenzoic acid were higher in the CK treatment compared to other treatments. Furthermore, *D-xylose* in RHB treatment and D-galactonic acid lactone in BOF treatment were at higher levels when compared to other treatments.

### 2.7. Plant Evaluation of Rice Growth Promotion

**Figure 5:**
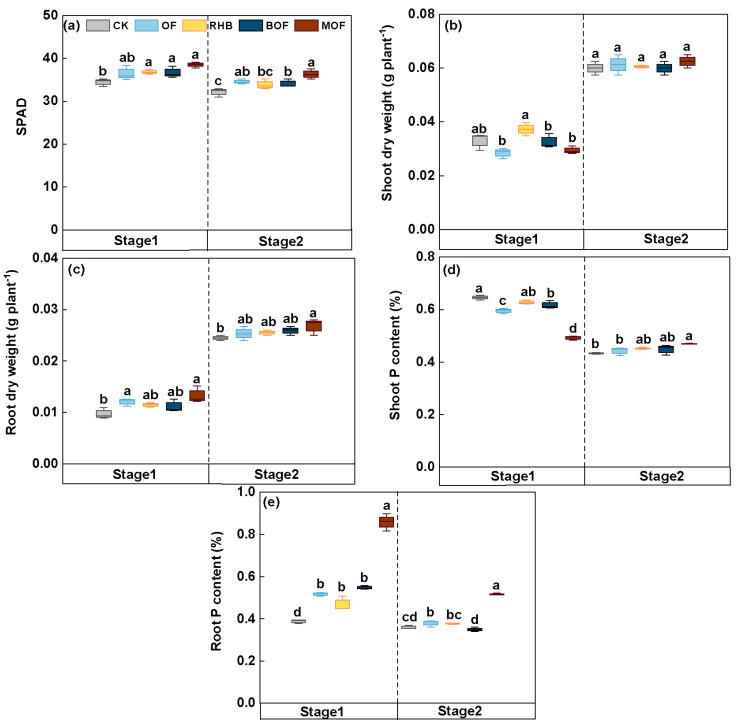
Rice growth parameters. (**a**) SPAD values (**b**) Shoot dry weight (**c**) Root dry weight (**d**) Shoot P content (**e**) Root P content. Stage 1 and Stage 2 represent days 21 and 38, respectively. Means ± standard deviations for three replicates. Different letters within a column indicate significant differences at *p* < 0.05. CK, control without fertilizer; OF; 0.1% organic fertilizer; RHB, 1% rice husk biochar; BOF, 1% rice husk biochar and 0.1% organic fertilizer. MOF, 1% rice husk BMB, and 0.1% organic fertilizer.

Phosphorus accumulation and plants’ dry weight were influenced differently by the various fertilization regimes. When compared with the control, SPAD values for all other treatments increased in two stages, and the MOF samples showed significant increases of 11.69% and 13.07%, respectively (*p* < 0.05) (Figure 5a). MOF treatment generated the highest values (0.469%) with significant increases of 8.31% on day 38 compared to the control (Figure 5d). In addition, the fertilization treatments had a positive effect on rice root growth. It can be seen that at 21 and 38 d, rice root dry weight and phosphorus content for MOF treatment were significantly (*p* < 0.05) higher than that of control and showed increases of 36.77 and 9.52% (1.21- and 0.43-fold), respectively (Figure 5c,e).

Compared to the control group, all treatments generated positive effects on root indices (Table S3). The total root length was significantly increased by 11.30–45.24% for the MOF treatment compared to the other treatments (*p* < 0.05). Furthermore, compared to the control group, the MOF treatment significantly increased root volume (23.2%), root projection area (33.16%), root surface area (33.20%) and the number of forks (43.68%) (*p* < 0.05). The above results show that the positive effect of MOF on root growth was demonstrated.

### 2.8. Relationships Among Soil Properties, Microbial Activity and Communities of pqqC and phoD

**Figure 6:**
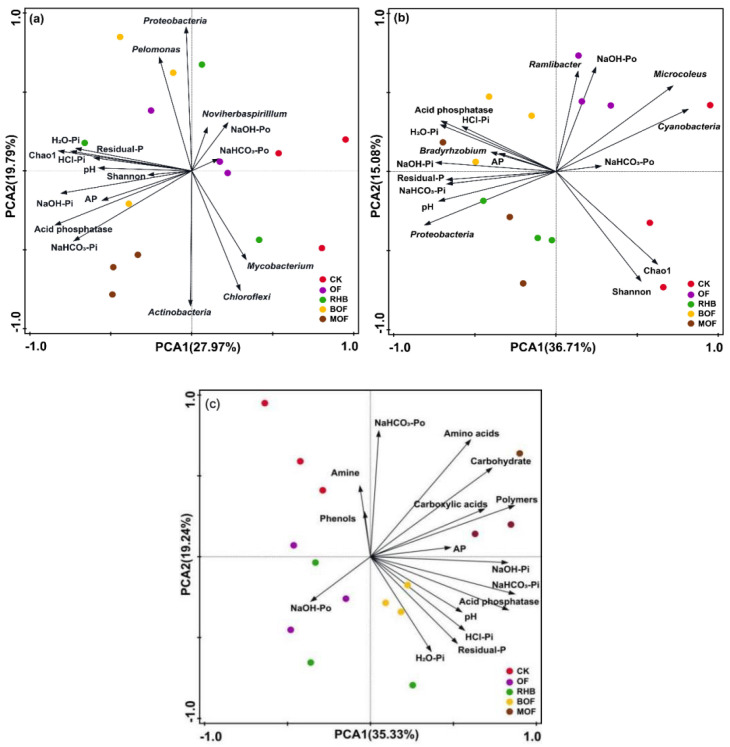
Principal component analysis (PCA) plots showing the relationships between soil properties and the diversity of the (**a**) *pqqC* and (**b**) *phoD* bacterial communities and (**c**) six groups of substrates (carbohydrates, carboxylic acids, polymers, phenols, amino acids, amines) after 96 h of incubation. Control, OF, RHB, BOF and MOF are indicated by red, purple, green, yellow and brown circles, respectively. The percentage of contribution is shown for each axis. CK, control without fertilizer; OF; 0.1% organic fertilizer; RHB, 1% rice husk biochar; BOF, 1% rice husk biochar and 0.1% organic fertilizer. MOF, 1% rice husk BMB and 0.1% organic fertilizer.

Principal component analysis (PCA) showed that PCA1 and PCA2 explained 27.97% and 19.79% of the variation in *pqqC* and 36.71% and 15.08% of the variation in *phoD*, respectively (Figure 6a,b). Control samples were mainly distributed in PCA1-positive regions, while different treatments caused the distribution of *pqqC* gene-carrying bacterial communities to shift to PCA1-negative regions (Figure 6a). Similarly, different treatments also caused the distribution of *phoD* gene-carrying bacterial communities to shift to PCA1-negative regions (Figure 6b). Figure 6c analysis was applied to reveal discriminations between various experimental treatments. The contributions of PCA1 and PCA2 accounted for 35.33 and 19.24%, respectively, to the variance of carbon source substrate. The control samples were primarily located in the negative zone of PCA1, while the different treatments caused a shift toward the positive zone. This analysis also revealed strong interrelations between soil properties such as pH, AP, and acid phosphatase, as well as carbohydrates, carboxylic acids, and polymers after 96 h of incubation.

### 2.9. Influential Factors on Rice Phosphorus Uptake

**Figure 7:**
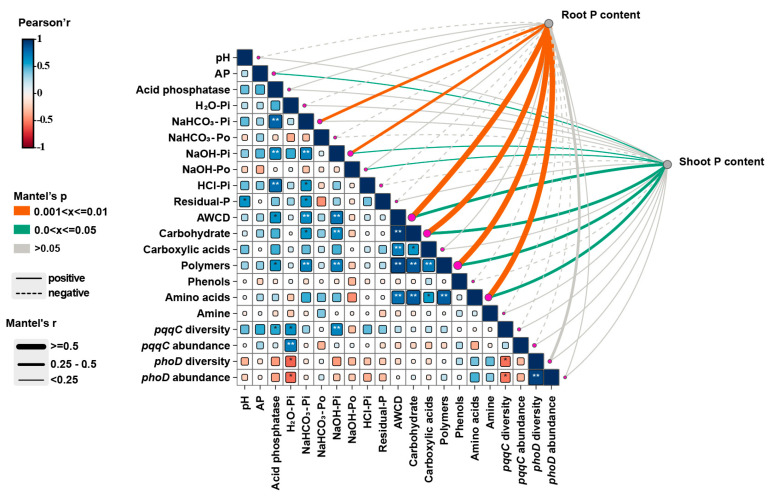
Pearson’s correlation matrix between variables under pot experiment(* *p* < 0.05, ** *p* < 0.01). The color density gradient and shape size represents the correlation coefficients, as indicated. The Mantel test was used to analyze the relationship between soil and plant variables and P contents in rice tissues. Line width corresponds to the Mantel R statistic, and line color relates to correlation.

We conducted the Mantel test to identify the factors affecting phosphorus uptake in rice plants (Figure 7). The shoot phosphorus content showed a significantly positive correlation with AP, NaOH–Pi, NaOH–Po, AWCD, carbohydrates, polymers, and amino acids (*p* < 0.05). Moreover, the root phosphorus content exhibited a highly significantly positive correlation with NaHCO_3_–Pi and NaOH–Pi, AWCD, carbohydrates, polymers, and amino acids (*p* < 0.01).

## 3. Discussion

### 3.1. Physico-Chemical Properties of BMB and Phosphorus Solubilization

Biochar is widely recognized as an excellent carrier material due to its porous structure [15]. It supports microbial habitats and contributes to facilitating PSB attachment and proliferation [20,21]. The SEM–EDS images of RHB and BMB presented here demonstrated its unique pore structure and chemical elements, which enabled the distribution of BM on the surface and within pores without altering the properties of the supporting biochar (Figure S1a,b). Meanwhile, the presence of Hydroxy, Carboxyl and Amine groups on biochar surfaces promotes bacterial adhesion as well as proliferation [17]. Thus, the dominant substances of biochar are available for PSB and affect the load of PSB.

BMB exhibited a high solubilization ability in growth media containing both organic and inorganic phosphorus, which had been attributed to phosphorus-solubilizing bacteria (BM) by secreting organic acids to reduce the pH of the media, thereby increasing the solubility of phosphorus (Figure S3) [22]. Additionally, biochar functions as a neutralizing agent, enhancing the acid production capacity of PSB and improving the adaptability of PSB to adverse environments [17,23]. It is worth noting that biochar contains functional groups such as Hydroxyls and Carboxyls, which actively contribute to phosphorus solubilization [24]. Therefore, the existence of BM promoted the P-solubilizing effect.

### 3.2. Soil Properties and Phosphorus Fractions

Low phosphorus availability is a key limiting factor for rice growth [25]. Numerous studies have demonstrated that biochar coupled with organic fertilizer significantly improved soil properties, including pH, nutrient storage and enzyme activities [18,26,27]. This is consistent with our research results, on the 21st day of the experiment, the pH and AP contents of MOF treatment were significantly increased compared with control, increasing by 8.16% and 44.74%, respectively. In our study, the application of biochar increased soil pH primarily due to the dissolution of alkaline substances (such as inorganic carbonate) in the biochar [28]. At the same time, the dynamic balance of active phosphorus, that is, the permanent exchange of dissolved phosphorus through the adsorption process, and the adsorbed phosphorus entering and leaving the soil solution according to factors such as pH value, temperature, humidity and concentration, determines the balance of phosphorus in the soil pores and the soil’s phosphorus buffer capacity [29,30]. Soil phosphorus availability tended to increase when the pH transitions from acidic conditions to a range of pH 6–7 (Table S2). For example, at the beginning of the experiment (day 0), soil pH in the RHB, BOF, and MOF treatment groups was significantly increased by 0.4 to 0.69 units compared to the control group, while available phosphorus (AP) content was also significantly increased by 18.59% to 49.74%. This is consistent with the results of the previous study [31]. The highest P content in MOF treatment indicated that PSB plays a role in increasing soluble phosphorus levels by producing organic acids, protons and hydroxide ions to dissolve minerals [6]. P may be readily soluble (e.g., a soluble compound like a phosphate fertilizer) or found in large organic molecules, which are subject to the rates of microbial organic matter decomposition [32]. Phosphatase activity also increased in all treatments compared with control, indicating high energy demand for microbes mobilizing organic phosphorus compounds [33]. Therefore, based on our findings, we speculate that the application of biochar coupled with organic fertilizer might stimulate the growth of PSB and subsequently promote AP release.

Phosphorus fractional analysis provides an additional method for investigating phosphorus transport and transformation in soils. Our results indicated that levels of H_2_O–Pi and NaHCO_3_–Pi for all treatments were increased, particularly for MOF (Figure 1a,b). Biochar application mainly impacts the soil’s inorganic phosphorus component, leading to increase phosphorus availability through concentration gradient diffusion [34]. Furthermore, the application of organic fertilizer might also increase soil organic carbon content, thereby reducing phosphorus adsorption and increasing desorption [8]. It is an important factor of P conversion in soil [35]. We observed a significant positive correlation between phosphatase activity and both NaHCO_3_–Pi and NaOH–Pi (Figure 7), which is consistent with the findings of Wang et al. [36]. The decrease in NaHCO_3_–Po and NaOH–Po content may be attributed to the hydrolysis of soil Po into Pi via extracellular phosphatases produced by BM [37]. Compared to the BOF treatment, a reduction in Residual–P content with the MOF treatment suggested that BM could promote the transition from stable to unstable phosphorus (Figure 4). This is mainly the conversion of insoluble P components into bioavailable phosphate by phosphate-solubilizing bacteria [38].

### 3.3. pqqC- and phoD-Harboring Bacterial Community

Biochar acts as a slow-release fertilizer and stores nutrients that promotes microbial growth [39]. In addition, it is possible that the inoculant could not survive better because of competition with native microorganisms [40], so the nutrients stored in biochar have a positive effect on increasing the community. Our results revealed that biochar application led to improvements in the diversity and abundance of *pqqC*-harboring bacteria compared with the control. BOF and MOF treatments significantly affected the Chao1 abundance index of the *pqqC* community, increasing by 55.69% and 50.25%, respectively (*p* < 0.05) (Figure S4b). The available organic carbon provided by biochar and organic fertilizers enters the soil environment, supplying external carbon for bacteria. This stimulation contributed to their growth and reproduction, ultimately enhancing bacterial diversity [8,41]. Notably, the diversity and abundance of *pqqC* in MOF treatment were lower than that in BOF treatment (Figure S4b). This difference is likely due to increased BM survival with biochar supports as it became dominant [12]. In contrast, the *phoD* diversity and abundance across various fertilization treatments were decreased. The Shannon diversity and Chao1 abundance indexes decreased by 3.38–12.29%, and 18.98–36.14%, respectively (Figure S4a,b), and this was similar to the findings of previous research [42]. It is possible that in low-nutrient soils, the absorption potential of *phoD*-harboring bacteria was inhibited due to competition for carbon sources and nutrients, leading to decreased abundance.

Consistent with previous studies, *Proteobacteria* and *Actinobacteria* were the dominant phyla in the *pqqC* and *phoD* populations [26,43]. At the same time, the result also indicated the importance of *Proteobacteria* and *Actinobacteria* in P-mobilizing microorganisms [8,44]. *Proteobacteria* and *Actinobacteria* are known as copiotrophic bacteria, and the application of biochar and organic fertilizer provided rich nutrients for stimulating growth and increasing their relative abundance, consistent with previous findings [26,41]. We found that *Bradyrhizobium* had a higher relative abundance, whereas soil AP content was higher. *Bradyrhizobium* is identified as the keystone flore in regulating the compositions and functions of the *phoD*-harboring communities [26] and produces phosphatase or organic acid to increase AP content [45]. Meanwhile, the presence of biochar and organic fertilizer might provide sufficient organic carbon supply for *Bradyrhizobium* survival. Among them, the abundance of *Bradyrhizobium* treated with BOF and MOF was as high as 41.40% and 38.54% (Figure 2d). Thus, OF and BMB applications promoted the formation of highly efficient P mobilization communities and enhanced their potential for Po mineralization and Pi solubilization.

### 3.4. Microbial Activity

In the present study, significant differences were observed in carbon source metabolism among different treatments. The microbial groups in the MOF treatment exhibited the highest capacity for carbon source utilization, as reflected by their high AWCD scores. After 96 h of incubation, MOF treatment showed a 4.70-fold increase in total AWCD compared to the control group (*p* < 0.05) (Figure 3a,b). Organic carbon serves as the primary source of microbial growth [8]. Meanwhile, the metabolism and reproduction of the soil microbial community can be promoted by increasing soil nutrients [46]. The addition of biochar and organic fertilizer in our study provided fresh exogenous organic carbon and nutrient elements for BM and phosphorus-mobilizing microorganisms were significantly positively correlated with multiple carbon sources (Figure 7).

The utilization of carbohydrates, carboxylic acids, polymers, and amino acid carbons was significantly increased by 3.12, 1.41, 5.95 and 0.86 units in the MOF treatment compared to the control (Figure S5). Although Carboxylic acid carbon sources constitute a minor fraction of soil organic matter, they serve as important C and N sources for microbial respiration and utilization, thereby contributing to soil nutrient accumulation [47,48]. Soil microorganisms obtain N from the soil organic matter to compensate for the high C: N ratio corrected by biochar, and this effect may explain the increased utilization of Amino acid substrates [49]. In addition, the addition of organic fertilizers or biochar both tended to reduce the utilization of phenolic acids (Figure 4). The increase in soil pH might contribute to the neutralization of Phenolic acids, thereby reducing the microbial utilization of phenolic acids [50]. PCA analysis indicated that the metabolic capacity of soil microorganisms in our control treatment was separate from that of other treatments (Figure 6c). This suggested that fertilization significantly altered the soil environment and consequently affected the characteristics of soil microorganism utilization of exogenous carbon sources. Thus, in the case of high utilization of carbon sources, bacterial metabolic activity was promoted to enhance the phosphorus solubilization ability of BM.

### 3.5. Effect on Rice Growth

The SPAD value is a well-established indicator for the measurement of leaf chlorophyll [51]. The highest SPAD content in MOF treatment proved that it promoted the growth and development of rice and significantly increased by 11.69% and 13.07% compared with the control in two stages (Figure 5). PSB may stimulate plant growth by regulating signaling pathways involved in photosynthesis [52]. Previous studies have widely reported the beneficial effects of biochar and organic fertilizer on soil nutrient enhancement and plant nutrient uptake [12,53]. Similarly, our results indicated that MOF treatment had a superior effect on plant phosphorus accumulation and phosphorus content was significantly increased by 9.52% compared with the control, suggesting BM inoculation produced the best effect on phosphorus mobilization (Figure 5). In addition, we observed an increase in both biomass and phosphorus content of rice roots relative to shoots. PSB inoculation was accompanied by microbe-induced changes in rice roots [2]. At the same time, biochar could increase soil porosity due to its unique pore structure [54], potentially allowing for greater root penetration. Compared with the control, the positive effect of MOF treatment on root parameters, significantly increasing root volume (23.2%), root protruding area (33.16%), root surface area (33.20%) and root branching number (43.68%) (Table S3). Increased uptake of phosphorus may promote root growth and thus increase nutrient uptake [55]. The maximum root diameter observed under MOF treatment (0.68 mm) aligns with the concept that thicker roots tend to anchor plants while enhancing nutrient transport capacity and promoting plant growth better [56].

## 4. Materials and Methods

### 4.1. Experimental Materials

The soil in this experiment was collected from the 0–20 cm depth of the surface layer of Xintong, Fuyang District, Hangzhou City, Zhejiang Province, Eastern China (119°83’ E, 29°91’ N). RHB was provided by Zhejiang Yangtze River Delta Junong Technology. The organic fertilizer (OF) utilized was a straw-mixed fertilizer made from rice straw. *Bacillus megaterium* was obtained from the BeNa Culture Collection and then cultured in a nutrient broth (NB) medium. The solid immobilized bacterial agent—BMB was obtained according to the method of Zhu et al. [57]. In brief, bacterial seed solution was produced by shaking at 160 rpm until logarithmic growth. Subsequently, 5 g of biochar that had been passed through a 100 mesh sieve was added into 100 mL NB and sterilized at 121 °C for 20 min. Then, the NB with inoculated bacterial seed solution (2%) was incubated in a shaker set at 160 rpm, 30 °C for 24 h. At last, the culture solution was subjected to centrifugation and washed with a saline solution containing a mass fraction of 1%. The physical and chemical properties of the soil, biochar, and organic matter utilized in this study are presented in Table S1.

### 4.2. Characterization and the Phosphate-Solubilizing Ability of BMB

Fourier transform infrared (FTIR, Thermo Fisher, Waltham, MA, USA) was performed on samples prepared by the KBr pressed-disk technique. The morphology and structure of RHB and BMB were examined using an SU8010 scanning electron microscope (SEM, Hitachi, Japan), and elemental content was determined by energy dispersive spectrometry (EDS).

Soluble phosphorus was quantified using 0.5 g of BMB inoculated into 100 mL Pi and Po media, with insoluble Ca_3_(PO_4_)_2_ and lecithin as the sole phosphorus source. The mixtures were then incubated at 30 °C with shaking at 160 rpm. Flasks lacking added materials or RHB were used as blank controls. After a 7-day incubation period, 2 mL samples were taken every 24 h, and the supernatant was extracted by centrifuging at 12,000 rpm for 10 min, repeated three times. Soluble phosphorus levels were determined using molybdenum blue colorimetry [18].

### 4.3. Pot Experiments and Sampling

Experimental soil samples were air-dried, ground through a 5 mm sieve, and added to plastic pots (85 × 85 × 108 mm) filled with 500 g of well-mixed air-dried soil. The fertilization regimes, including three replicates of five treatments, were arranged in a completely randomized design. The treatments consisted of: (1) control without fertilizer (CK), (2) organic fertilizer (OF), (3) rice husk biochar (RHB), (4) rice husk biochar and organic fertilizer combined (BOF), as well as (5) rice husk BMB and organic fertilizer combined (MOF). OF, RHB, and rice husk BMB were added at the ratio of 0.1%, 1%, and 1% of soil weight, respectively. The rice seeds (cultivar Shenchengyou 913) were germinated in tap water. After growing for 7 days, they were gently collected and then 10 healthy seeds with similar height were selected for transplantation. The seeds were planted in equal spaces apart from each other in the pot with a maintained water layer of about 2 cm. The experiment was started on 24th October 2023, and top dressing N (20 mg kg^−1^ soil) and K (50 mg kg^−1^ soil) were applied as (NH_4_)_2_SO_4_ and KCl, respectively. It was incubated under outdoor temperature conditions and flooded throughout the experiment to simulate the situation of flooded rice. Destructive sampling was conducted at 0, 21, and 38 d after the start of the experiment. A portion of each fresh soil sample was stored at −80 °C, while the remainder was air-dried and passed through 10- and 100-mesh sieves. The plants were then rinsed with deionized water and divided into above-ground (shoot) and below-ground (root) portions. Subsequently, they were dried at 70 °C until constant weight and finely ground for analysis.

### 4.4. Sample Testing and Soil Phosphorus Fractionation

Soil pH was measured using a pH meter with a soil-to-water ratio of 1:2.5 (m/V). Total phosphorus (TP) was determined by digestion with perchloric acid (HClO_4_), and AP was determined following 0.5 M NaHCO_3_ extraction as previously described [58]. Soil acid phosphatase activity was determined by phenylene disodium phosphate colorimetry [59]. Soil phosphorus fraction content was determined using the Hedley sequential extraction procedure modified as previously reported [60]. Briefly, 0.5 g soil was extracted in sequence using 30 mL (0.5 M NaHCO_3_ pH 8.5, 0.1 M NaOH and 1.0 M HCl) with oscillation for 16 h at 25 °C at 180 rpm. Supernatants of centrifugation were digested with acidified ammonium persulfate. Po was calculated as the difference between total P (Pt) and Pi. Finally, the soil residue was digested with H_2_SO_4_–H_2_O_2_ to extract residual phosphorus. Phosphorus levels in all extracts and digestive fluids were determined. Fresh plant roots were analyzed using an Epson V 700 scanner with WinRHIZO Pro software (Reagent Instruments, Québec City, QC, Canada). Chlorophyll levels in shoots were measured using a Soil and Plant Analyzer Development (SPAD) 502 Plus meter. The dried plant samples were digested with H_2_SO_4_–H_2_O_2_ and phosphorus content in the solution was determined with the molybdenum ascorbate blue method [58].

### 4.5. High-Throughput Sequencing and Data Processing

Genomic DNA was extracted from freeze-dried soil using the MagaBi Soil/Stool DNA Kit (Bioer, Hangzhou, China) according to the manufacturer’s instructions. The purity of DNA was determined using a Qubit 4.0 instrument (Thermo Fisher Scientific, Waltham, MA, USA). The amplification primers for bacterial *phoD* and *pqqC* genes were as follows: (5’ to 3’) CAGTGGGACGACCACGAGGT/GAGGCCGATCGGCATGTCG) [61] and AACCGCTTCTACTACCAG/GCGAACAGCTCGGTCAG) [62]. The PCR amplification protocol was as follows: 94 °C for 5 min, 30 cycles of 94 °C for 30 s, 52 °C for 30 s, 72 °C for 30 s and 10 min at 72 °C. PCR amplicons were purified using the BSC48L1E-G DNA Kit (Bioer, Hangzhou, China). The purified amplicons were pooled in equimolar proportions and sequenced using the PE250 method on an Illumina Nova 6000 platform at Guangdong Magigene Biotechnology (Guangzhou, China). Sequencing data generated in this study have been deposited at the NCBI Sequence Read Archive under accession code SRP542393.

The software fastp (version 0.14.1) was used for clipping and primer removal from the raw data to obtain clean reads. Operational taxonomic units (OTUs) were defined with a 97% similarity cutoff using USEARCH. The community composition and abundance were analyzed using R microeco package (v1.12.0). Diversity indices were calculated based on OTUs using usearch-alpha_div (V10, http://www.drive5.com/usearch/ (accessed on 14 March 2024).

### 4.6. Biolog EcoPlates Assays of Soil Microbiota

The soil microbiota in each soil sample was analyzed using Biolog EcoPlates (Hayward, CA, USA) containing 31 different carbon sources to examine the relative carbon utilization in bacterial populations. Briefly, 10 g fresh soil was mixed with 100 mL 0.05 M pH 7 sterilized Phosphate Buffered Saline (PBS) and oscillated for 30 min. Thereafter, 1 mL of the supernatant was diluted using sterilized PBS (1×) up to 10^−3^-fold. Subsequently, 150 μL diluted suspension was added to each well of the Biolog EcoPlate, and the microplate was incubated at 25 °C for 168 h. The optical density (OD) value of each well on the Biolog EcoPlate was measured at a wavelength of 590 nm every 12 h. Soil microbial activity was calculated as the average well-color development (AWCD), which is determined by using the following equation:

A = ∑(Ci − R)/31(1)

where Ci represents the OD value of each sample well, and R is the OD value of the control well.

### 4.7. Data Analysis

The data manipulation and statistical analysis were conducted using Microsoft Excel and SPSS 27.0. A one-way analysis of variance (ANOVA) with the Duncan test was performed to analyze the data, with the criterion for statistical significance set at *p* < 0.05 to determine the significant differences in physicochemical parameters among different treatments. The results were visualized using Origin 2024. Principal component analysis (PCA) was carried out using Canoco 5.0 to reveal relationships between soil properties, phosphorus fractions and microbial indicators. Pearson’s correlation coefficient was employed to analyze the relationships among soil and plant variables. Heat maps were generated using ChiPlot (https://www.chiplot.online (accessed on 14 March 2024)).

## 5. Conclusions

Due to the limited availability of phosphorus in rice cultivation, the development of an efficient biochar-based fertilizer could achieve greater benefits. Our study indicated that soil phosphorus conversion significantly increased AP for BMB and OF combined while decreasing levels of inactive phosphorus. Simultaneously, the application of BMB and OF greatly reshaped the community structure of phosphorus-mobilizing bacteria and significantly increased microbial catabolic activity, as indicated by the highest values of AWCD. This was particularly evident in the utilization of carbohydrates, carboxylic acids, and amino acid carbons. It was confirmed that the application of BMB and OF promoted phosphorus uptake and growth in rice. Hence, this study provides a theoretical basis for achieving high yields in rice production. As well as Experimental evidence showing adsorption or desorption behavior under controlled conditions would help elucidate the role of soil particles and biochar in phosphorus dynamics.

## Data Availability

Data will be made available on request.

## Supplementary Materials

The following supporting information can be downloaded at: https://www.mdpi.com/article/10.3390/plants14020214/s1, Table S1. Physicochemical properties of the experimental soil, biochar and organic fertilizer. Table S2. Effects of different fertilization treatments on soil at 0, 21, 38 days. Table S3. Morphological characteristics of rice root systems at 38 days post-treatment. Figure S1. SEM and EDS images of RHB (a, b) and BMB (c, d). RHB, rick husk biochar; rice husk biochar-immobilized *Bacillus megaterium* Morphological characteristics of rice root systems at 38 days post-treatment. Figure S2. FTIR images of RHB and BMB. RHB, rick husk biochar; rice husk biochar-immobilized *Bacillus megaterium*. Figure S3. Phosphorus solubilizing capacity of BMB in different phosphorus medium. Means ± standard deviations for three replicates. Figure S4. Diversity and abundance of the *pqqC* and *phoD*-harboring bacterial community. Means ± standard deviations for three replicates. Different letters within a column indicate significant differences at *p* < 0.05. CK, control without fertilizer; OF; 0.1% organic fertilizer; RHB, 1% rice husk biochar; BOF, 1% rice husk biochar and 0.1% organic fertilizer. MOF, 1% rice husk biochar-immobilized *Bacillus megaterium* and 0.1% organic fertilizer. Figure. S5. Utilization of six major carbon source after 96 hours under different treatments. Means ± standard deviations for three replicates. Different letters within a column indicate significant differences at *p* < 0.05. CK, control without fertilizer; OF; 0.1% organic fertilizer; RHB, 1% rice husk biochar; BOF, 1% rice husk biochar and 0.1% organic fertilizer. MOF, 1% rice husk biochar-immobilized *Bacillus megaterium* and 0.1% organic fertilizer.
