# Supplementary material for: Application of Biochar-Immobilized Bacillus megaterium for Enhancing Phosphorus Uptake and Growth in Rice
Source: Plants (Basel). 2025 Jan 14;14(2):214. doi: 10.3390/plants14020214 (PMC11768149; doi:10.3390/plants14020214)
Supplement: Supplementary file 1 [file plants-14-00214-s001.zip › plants-3342670-supplementary.pdf]

Supplementary Material

Table S1 Physicochemical properties of the experimental soil, biochar and organic fertilizer.

	Soil	Rice husk biochar	Organic fertilizer
pH	5.64	8.10	8.96
Ash (%)	-	45.00	-
TN (%)	-	0.37	0.16
TP (%)	0.49	0.18	0.41
TK (%)	-	0.74	1.43
SOC (%)	0.82	49.25	27.47
AN (mg/kg)	76.37	-	-
AP (mg/kg)	7.64	-	-
AK (mg/kg)	60.67	-	-

[1] TN: total nitrogen; TP: total phosphorus; TK: total K; SOC: soil organic carbon; AN: alkali-hydrolyzable nitrogen; AP: available phosphorus; AK: available potassium.

Table S2 Effects of different fertilization treatments on soil at 0, 21, 38 days.

Treatment	Day0			Day21			Day38		
	pH	AP (mg/kg)	TP (g/kg)	pH	AP (mg/kg)	Acid phosphatase (mg $g^{-1}\cdot$24h)	pH	AP (mg/kg)	Acid phosphatase (mg $g^{-1}\cdot$24h)
CK	5.64±0.03e	7.64±0.07c	0.49±0.01c	6.13±0.07bc	9.99±0.92c	0.386±0.03b	6.01±0.09b	6.41±0.59a	0.229±0.02b
OF	5.84±0.03d	8.87±1.00b	0.51±0.01c	6.07±0.05c	14.40±0.30a	0.557±0.03a	5.97±0.07b	6.92±0.40a	0.254±0.01ab
RHB	6.33±0.04a	10.99±0.67a	0.55±0.06b	6.20±0.42b	12.75±0.53b	0.370±0.022b	6.24±0.05a	6.94±1.08a	0.252±0.035ab
BOF	6.13±0.04b	11.44±0.59a	0.55±0.01b	6.23±0.04b	10.34±0.59c	0.428±0.10b	6.25±0.05a	6.71±1.02a	0.264±0.01ab
MOF	6.04±0.02c	9.06±0.65b	0.59±0.03a	6.63±0.12a	14.46±0.83a	0.417±0.02b	6.19±0.03a	7.83±0.51a	0.282±0.02a

[1] Means ± standard deviations for three replicates. Different letters within a column indicate significant differences at $P < 0.05$. CK, control without fertilizer; OF; 0.1% organic fertilizer; RHB, 1% rice husk biochar; BOF, 1% rice husk biochar and 0.1% organic fertilizer. MOF, 1% rice husk biochar-immobilized *Bacillus megaterium* and 0.1% organic fertilizer.

Table S3 Morphological characteristics of rice root systems at 38 days post-treatment.

Treatment	Total root length (cm)	AvgDiam (mm)	Root volume (cm^3)	Projection area (cm^2)	Surface area (cm^2)	No. of tips	No. of forks	No. of cross
CK	136.08±8.03c	0.60±0.07a	0.50±0.08b	9.86±0.51c	30.96±1.63c	904±137a	1037±83b	137±38b
OF	177.57±2.09b	0.63±0.02a	0.61±0.02ab	11.71±0.11b	36.80±0.36b	930±23a	1203±66ab	215±27a
RHB	166.94±5.29b	0.63±0.01a	0.52±0.04b	10.64±1.12bc	32.97±1.64c	977±64a	1256±74ab	171±29ab
BOF	173.13±13.08b	0.61±0.05a	0.51±0.05b	10.62±1.21bc	33.36±3.79bc	930±51a	1470±286a	223±33a
MOF	197.64±14.32a	0.68±0.04a	0.69±0.04a	13.13±0.14a	41.24±0.45a	1076±168a	1490±302a	214±58a

[1] Means ± standard deviations for three replicates. Different letters within a column indicate significant differences at $P < 0.05$. CK, control without fertilizer; OF; 0.1% organic fertilizer; RHB, 1% rice husk biochar; BOF, 1% rice husk biochar and 0.1% organic fertilizer. MOF, 1% rice husk biochar-immobilized *Bacillus megaterium* and 0.1% organic fertilizer.

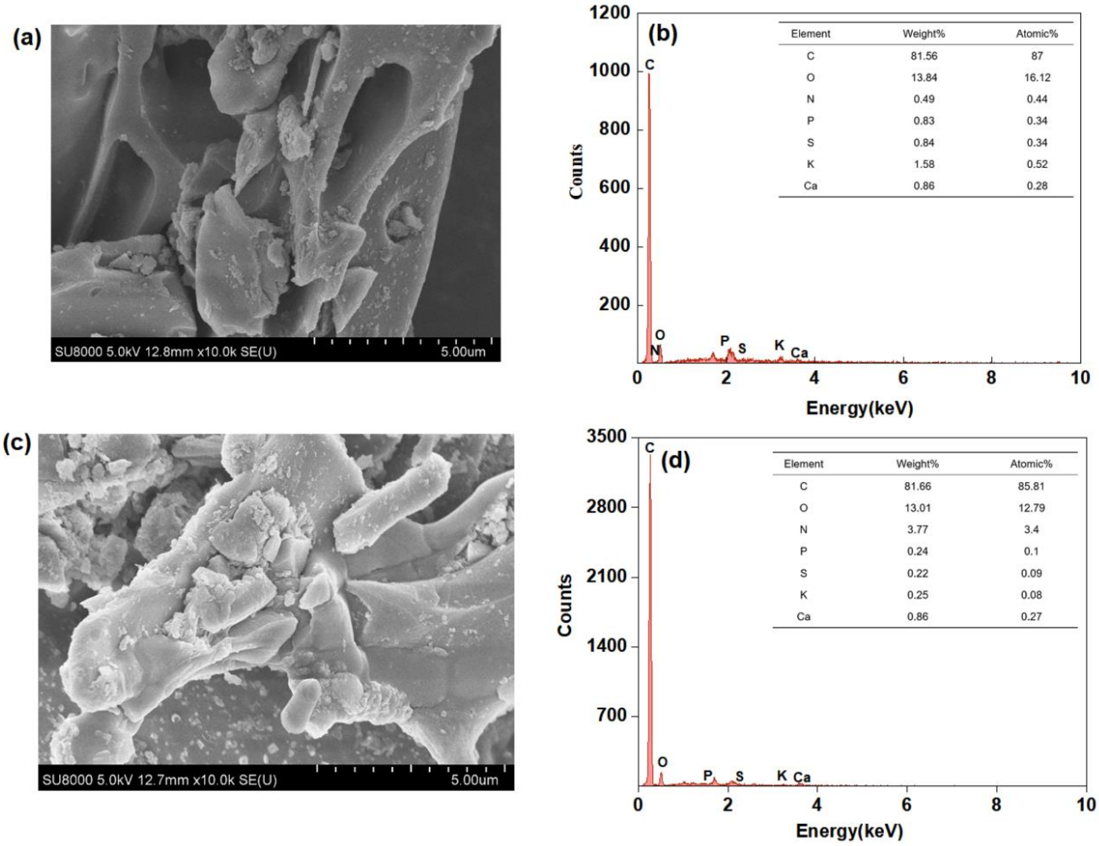

Figure. S1. SEM and EDS images of RHB (a, b) and BMB (c, d). RHB, rick husk biochar; rice husk biochar-immobilized *Bacillus megaterium*

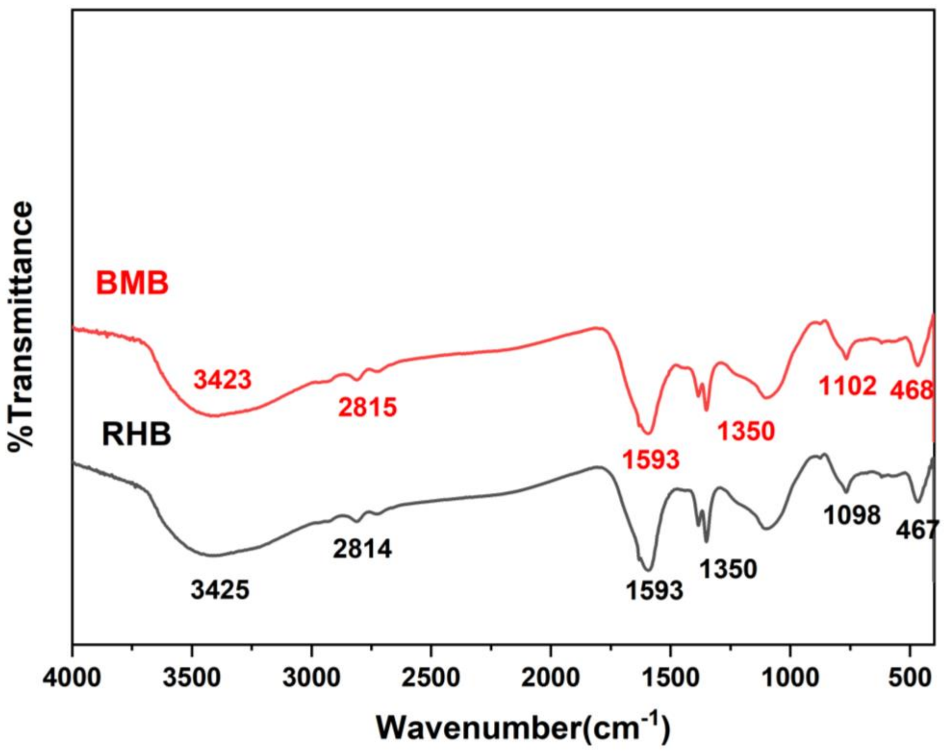

Figure. S2. FTIR images of RHB and BMB. RHB, rick husk biochar; rice husk biochar-immobilized *Bacillus megaterium*

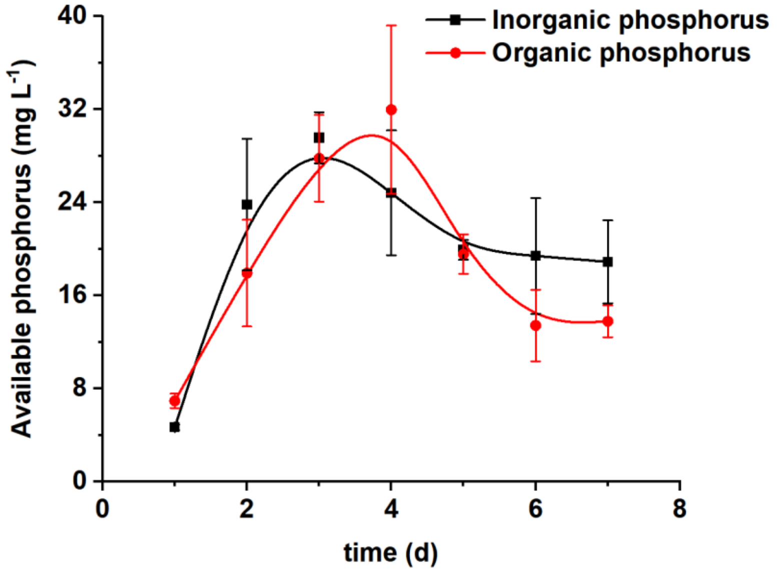

Figure. S3. Phosphorus solubilizing capacity of BMB in different phosphorus medium. Means ± standard deviations for three replicates.

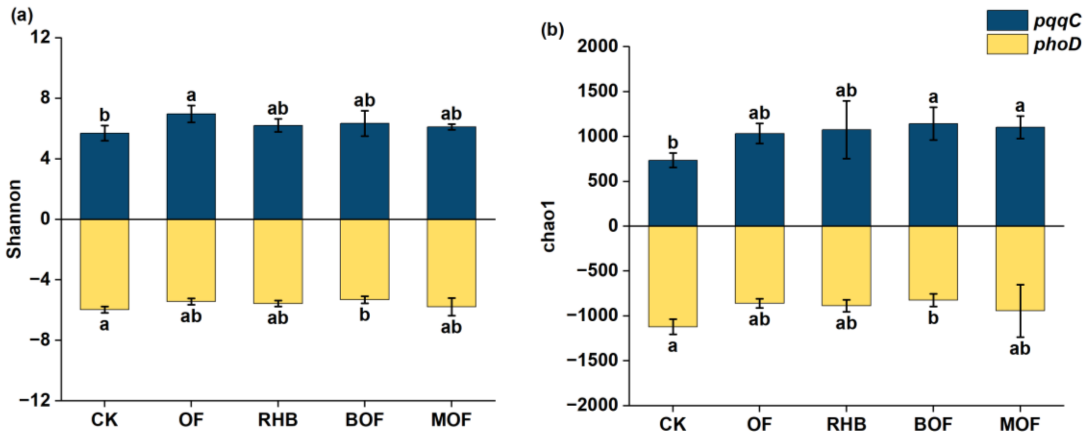

Figure. S4. Diversity and abundance of the *pqqC* and *phoD*-harboring bacterial community. Means ± standard deviations for three replicates. Different letters within a column indicate significant differences at $P < 0.05$. CK, control without fertilizer; OF; 0.1% organic fertilizer; RHB, 1% rice husk biochar; BOF, 1% rice husk biochar and 0.1% organic fertilizer. MOF, 1% rice husk biochar-immobilized *Bacillus megaterium* and 0.1% organic fertilizer.

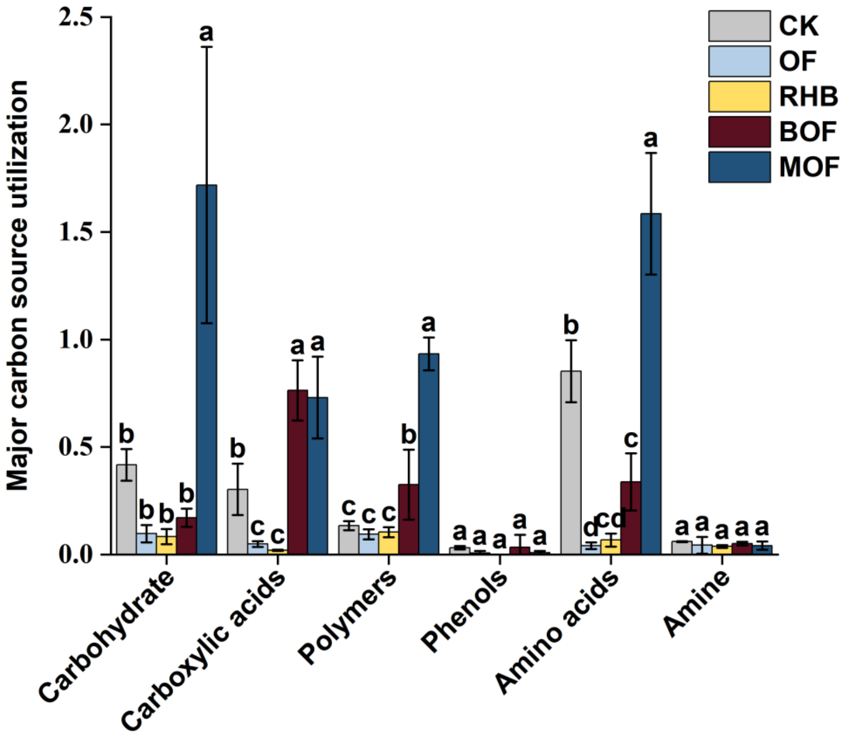

Figure. S5. Utilization of six major carbon source after 96 hours under different treatments. Means ± standard deviations for three replicates. Different letters within a column indicate significant differences at $P < 0.05$. CK, control without fertilizer; OF; 0.1% organic fertilizer; RHB, 1% rice husk biochar; BOF, 1% rice husk biochar and 0.1% organic fertilizer. MOF, 1% rice husk biochar-immobilized *Bacillus megaterium* and 0.1% organic fertilizer.
